# Long-Term Dynamics of Bluetongue Virus in Wild Ruminants: Relationship with Outbreaks in Livestock in Spain, 2006-2011

**DOI:** 10.1371/journal.pone.0100027

**Published:** 2014-06-18

**Authors:** Cristina Lorca-Oró, Jorge Ramón López-Olvera, Francisco Ruiz-Fons, Pelayo Acevedo, Ignacio García-Bocanegra, Álvaro Oleaga, Christian Gortázar, Joan Pujols

**Affiliations:** 1 Centre de Recerca en Sanitat Animal (CReSA), UAB-IRTA, Campus de la Universitat Autònoma de Barcelona, Bellaterra (Cerdanyola del Vallès), Spain; 2 Servei d’Ecopatologia de Fauna Salvatge (SEFaS), Departament de Medicina i Cirurgia Animals, Universitat Autònoma de Barcelona (UAB), Bellaterra, Barcelona, Spain; 3 SaBio IREC, Instituto de Investigación en Recursos Cinegéticos (CSIC-UCLM-JCCM), Ronda de Toledo s.n., Ciudad Real, Spain; 4 CIBIO, Centro de Investigação em Biodiversidade e Recursos Genéticos, InBio Laboratório Associado. Universidade do Porto, Vairão, Portugal; 5 Departamento de Sanidad Animal, Facultad de Veterinaria, UCO, Campus Universitarios de Rabanales, Córdoba, Spain; 6 SERPA, Sociedad de Servicios del Principado de Asturias, S.A., Gijón, Asturias, Spain; 7 Institut de Recerca i Tecnologia Agroalimentàries (IRTA), Barcelona, Spain; The Pirbright Institute, United Kingdom

## Abstract

Wild and domestic ruminants are susceptible to Bluetongue virus (BTV) infection. Three BTV serotypes (BTV-4, BTV-1 and BTV-8) have been detected in Spain in the last decade. Even though control strategies have been applied to livestock, BTV circulation has been frequently detected in wild ruminant populations in Spain. The aim of the present study is to assess the role for wild ruminants in maintaining BTV after the vaccination programs in livestock in mainland Spain. A total of 931 out 1,914 (48.6%) serum samples, collected from eight different wild ruminant species between 2006 and 2011, were BTV positive by ELISA. In order to detect specific antibodies against BTV-1, BTV-4 and BTV-8, positive sera were also tested by serumneutralisation test (SNT). From the ELISA positive samples that could be tested by SNT (687 out of 931), 292 (42.5%) showed neutralising antibodies against one or two BTV serotypes. For each BTV seroptype, the number of outbreaks in livestock (11,857 outbreaks in total) was modelled with pure autoregressive models and the resulting smoothed values, representing the predicted number of BTV outbreaks in livestock at municipality level, were positively correlated with BTV persistence in wild species. The strength of this relationship significantly decreased as red deer (*Cervus elaphus*) population abundance increased. In addition, BTV RNA was detected by real time RT-PCR in 32 out of 311 (10.3%) spleen samples from seropositive animals. Although BT outbreaks in livestock have decreased substantially after vaccination campaigns, our results indicated that wild ruminants have been exposed to BTV in territories where outbreaks in domestic animals occurred. The detection of BTV RNA and spatial association between BT outbreaks in livestock and BTV rates in red deer are consistent with the hypothesis of virus circulation and BTV maintenance within Iberian wild ruminant populations.

## Introduction

Bluetongue (BT) is a vector-borne infectious disease that has geographically expanded in Europe during the last decades [1]–[5]. The causal agent, bluetongue virus (BTV), is transmitted by *Culicoides* biting midges and both wild and domestic ruminants and camelids are considered susceptible hosts. Vector and host density, as well as environmental factors, are implicated in the distribution of BT, which is considered endemic in wild ruminants in large parts of Africa and North America [6], [7]. Except for mouflon (*Ovis aries musimon*), European wild ruminants are mostly asymptomatic to BTV infection [8]–[10], and they have potential to participate in BTV epidemiology [11]–[14]. However, the role of European wild ruminants in BTV transmission and maintenance is still under debate [15]–[17].

In Europe, BTV serotype 4 (BTV-4) was firstly detected in 2004 in Southern Spain and was detected in livestock until the end of 2007. Spain was declared free from BTV-4 in 2009, but this serotype reappeared in 2010. BTV-1 was detected in livestock from Southern Spain in 2007, and as with BTV-4, both BTV strains reached the Iberian Peninsula probably from infected *Culicoides* carried by the wind from North Africa [18], and spread in the following years to northern areas. BTV-8 appeared in Central Europe in 2006 and reached Northern Spain in 2008. Vaccination against BTV-4 was compulsory in susceptible domestic ruminants from 2005 to 2008, when the epidemiological situation of BTV-4 in Spain was thought to be controlled, but in 2010 a new compulsory vaccination campaign against this serotype was implemented. In 2007 and in 2008, compulsory vaccination campaigns were implemented in Spain for BTV-1 and BTV-8, respectively. Vaccination against BTV-4, BTV-1 and BTV-8 serotypes was compulsory until 2011 [19]. Spain is currently considered free from BTV-8 and continues to be considered a restriction zone for BTV-1 and BTV-4, the latter only in Southern Spain [19].

BTV specific antibodies have been detected both in free-ranging and farmed wild ruminants in several European countries [20]–[24] and BTV RNA has been detected in wild free-ranging ruminants [12], suggesting BTV circulation in these species. In Spain, BTV specific antibodies and BTV RNA have also been found in wild ruminants [10], [11], [13], [14], [16], [17], [25], [26] and susceptibility to BTV infection has been experimentally demonstrated both in red deer (*Cervus elaphus*) and the endemic Spanish ibex (*Capra pyrenaica*) [27], [28].

In order to understand the role of wildlife in BTV epidemiology in Spain, the aim of this study is to analyze the long-term dynamics, transmission and maintenance of BTV serotypes 1, 4 and 8 in wild ruminants during the period 2006–2011, as well as their relationship with BTV outbreaks in domestic ruminants.

## Materials and Methods

### Ethics Statement

This study did not involve purposeful killing of animals. Professional personnel collected blood and spleen samples mostly from hunted-harvested wild ruminants during the hunting seasons. These animals were legally hunted under Spanish and EU legislation and all hunters had hunting licenses. When possible, blood samples were collected from live-farmed individuals. No ethical approval was deemed necessary; the collection of all the samples was performed for routine procedures before the design of this study in compliance with the Ethical Principles in Animal Research. Thus, blood or spleen samples were not collected specifically for this study. Protocols, amendments and other resources were done according to the guidelines approved by each Autonomous government following the R.D.1201/2005 of the Ministry of Presidency of Spain (10th October 2005, BOE 21st October 2005) (http://www.umh.es/_web_rw/ceie/docs/animales/1201_05%20proteccion%20animales%20experimentacion.pdf).

### Samples

**Figure 1:**
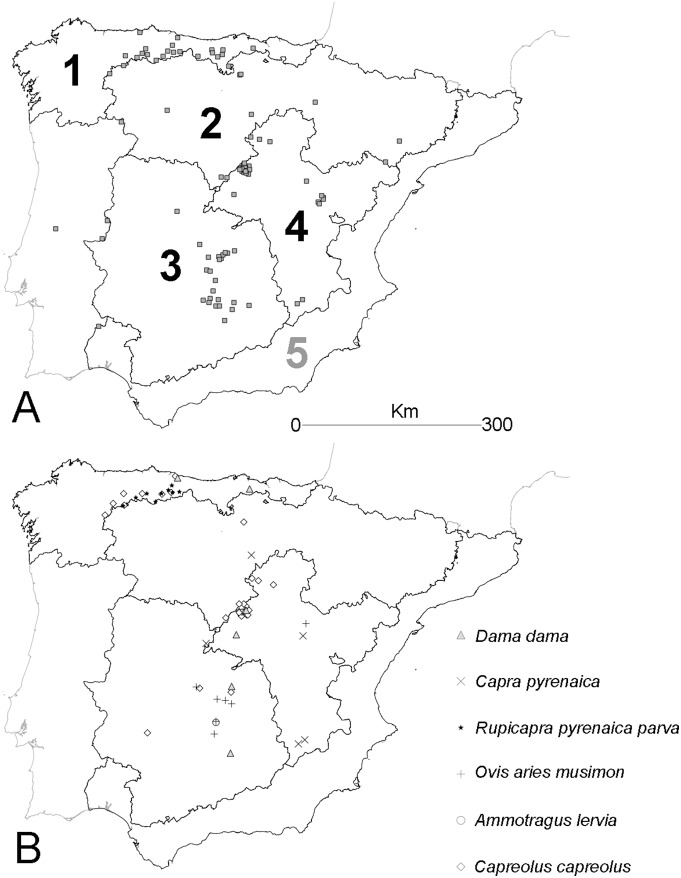
Distribution of the wild ruminant sampled populations (A: red deer; B: other wild ruminant species) and bio-regions in Spain: (1) Atlantic, (2) Northern Plateau, (3) South-Central, (4) Interior Mountains (adapted from [46]; see also [47]).

Samples were collected in Spain from 90 wild ruminant populations grouped according to bio-regions (from 1 to 4, region number 5 was not sampled due to scarce presence of wild ruminants; see Figure 1) considered by the Spanish Wildlife Disease Surveillance Scheme [29].

A total of 1,914 serum samples from 1,356 red deer, 227 fallow deer (*Dama dama*), 130 roe deer (*Capreolus capreolus*), 131 Cantabrian chamois (*Rupicapra pyrenaica parva*), 36 Spanish ibex, 31 European mouflon and three aoudad (*Ammotragus lervia*) were collected between March 2006 and June 2011. Samples assigned to each year were those obtained from April to March of the following year, according to the theoretical vector presence, as used in former studies [23], [30]. Animals were classified in five age groups according to tooth eruption patterns [31]: i) yearling (0–12 months old); ii) juvenile (1–2 years old); iii) sub-adult (2–3 years old); and iv) adult (>4 years old). Blood samples from the wild ruminants used in this study were collected into sterile tubes without anticoagulant either by jugular venipuncture from live animals or from the heart or thoracic cavity of legally hunter harvested animals. Sera were obtained after centrifugation at 300×G for 15 minutes and stored at −20°C until analysis. In addition, spleen samples were obtained from 311 individuals also included in the serological tests. Spleen samples were kept at −80°C until analysis.

### Serological Analyses

All serum samples were tested for the presence of BTV specific antibodies against the major core protein VP7 using a commercial double-antigen ELISA assay (Ingezim BTV DR12.BTV.KO, Ingenasa, Spain), according to manufacturer’s instructions.

ELISA positive sera were further analyzed by serum neutralisation test (SNT) to detect BTV-1, BTV-4 and BTV-8 specific neutralising antibodies, as previously reported [32]. Briefly, heat inactivated sera (56°C for 30 minutes) were diluted from 1∶2 to 1∶4096 in microplates (Costar Cat. N° 3915, Cultek, Madrid, Spain) using MEM Earle (Eagle’s minimum essential medium with Earle salts) and mixed with 100 TCID_50_ of each reference strain (BTV-1/ALG/2006, BTV-4/SPA/2004 and BTV-8/BEL/2006). Mixtures were incubated for one hour at 37°C, and 100 µL of a VERO E6 cell suspension in MEM supplemented with 15% foetal bovine serum (FBS; PAA Laboratories GmbH, Austria), 300 µg/l-glutamine/mL, 300 U penicillin/mL and 300 µg streptomycin/mL, were added to a final concentration of 1.5×10^4^/well. The mixture was further incubated for six days at 37°C, plate readings for cytopathic effect (CPE) were done at four and six days. Developing CPE was compared with control wells containing 100 TCID_50_ of virus and negative control wells (without virus). Only samples that showed neutralisation (absence of CPE) at dilutions ≥1∶4 were considered positive.

### BTV RNA Detection

Spleen samples from 311 ELISA positive individuals were analyzed to detect BTV RNA. Previously, total RNA was extracted using the Biosprint 96 kit (Qiagen, Germany). RT-qPCR was performed using the primers and the specific probe for BTV segment 5 as previously described [33]. Amplification of BTV was carried out using an AgPath-IDTM One-Step RT-PCR kit (Applied Biosystems, UK) in 7500 Fast equipment using 2 µL of eluted RNA in a total volume of 20 µL. According to the National BTV Reference Laboratory in Algete (Madrid), reactions were carried out using an amplification program consisting of an initial denaturing step at 95°C for 5 minutes and the following cycling conditions: 48°C for 10 minutes, 95°C for 10 minutes and 40 cycles at 97°C for 3 seconds and 61°C for 30 seconds.

### Spatial Trends in BTV Outbreaks in Domestics

In order to explore the relationship between BTV detection in wild ruminants and BTV circulation in domestic livestock, the spatial trends of BT outbreaks caused by BTV-1, BTV-4 and BTV-8 in livestock during 2003–2012 were analysed at municipality level. Information for 11,857 outbreaks in livestock affecting more than 1,300 municipalities were analysed [19]: BTV-1 outbreaks were detected in 1,219 municipalities, BTV-4 in 142, and BTV-8 in only 14 municipalities. The absence of a temporal element in the spatial models was due to sampling size limitations for estimating reliable annual BTV prevalence for each wild population.

For each BTV serotype, the total number of livestock outbreaks per municipality was modelled (until December 2012) using spatial autoregressive models developed with Spatial Analyses in Macroecology software [34]. The autoregressive models were not used to explain the pattern of BTV outbreaks in domestic animals in relation to risk factors (e.g. livestock abundance, climate, vector abundance, etc.), but to extract relevant spatial effects present in the data while simultaneously removing extreme values [35]. The resulting smoothed patterns for the number of BT outbreaks increased precision and specificity without introducing significant bias [36], and thus they were used as a proxy of BTV transmission risk to wild ungulates. As outbreak data were referred to municipalities, the uncertainty of the local measurement and the spatial dependence between neighbouring measurements was taken into account by using smoothing procedures [37]. In this study the neighbours for a target municipality were those closer than 30 km. The selection of this threshold was based on the high variation in size of the Spanish municipalities (from 0.02 to 1750 km^2^), that recommended against the use of relative neighbours, and on exploratory analysis considering several distance thresholds (namely, 5, 10, 15, 20, 25, and 30 km) according to previous studies [38]. Finally, 30 km was the threshold that produced more coherent predictions and warranted that all municipalities had at least one neighbour [39]. The response variables in these pure autoregressive models were the number of BT outbreaks for each BTV serotype at municipality level, and they were modelled by considering the spatial structure of neighbours. As a fundamental assumption of the models, the spatial independence of the residual values was assessed with Moran’s I test. Finally, the R^2^ was used as a measure of the fit of the models.

### Statistical Analysis

Associations between BTV seroprevalence determined by ELISA in all wild ruminant species and independent factors (species, sampling period, sex, age, and bio-region) were analyzed using a Pearson’s chi-square test. When observations per category were less than six, Fisher’s exact test was used. Differences between categories were analyzed by Tukey tests.

Generalized linear models (binomial distribution and link logit; [40]) were used to assess the association between BTV predicted outbreaks in livestock and BTV prevalence determined by SNT in wild ruminants. The reduced number of municipalities with BTV-4 and BTV-8 outbreaks in domestic livestock, and the number and distribution of wild ruminant samples (Table 1, Figure 1), made analysis between BT outbreaks in livestock and BTV prevalence in wild ruminants only feasible for BTV-1 and red deer. Thus, for each wild ruminant sampled population the number of outbreaks of each BTV serotype predicted by the autoregressive models was extracted in a buffer area around populations. The size of the buffer area was fitted to 5 km according to the ecology of both vectors and red deer [30], [41]. The response variable in this analysis was the number of BTV-1 seropositive red deer in each sampled population in relation to sample size, and the number of predicted BTV outbreaks in domestic livestock; the relative red deer abundance [30] and their interaction, were included as predictors.

**Table 1:** Number of BTV positive/analyzed (P/N) individuals and percentage of positive sera (95% CI) by ELISA stratified by species and sampling period.

	2006–2007		2007–2008		2008–2009		2009–2010		2010–2011		Total	
Species	P/N	%	P/N	%	P/N	%	P/N	%	P/N	%	P/N	%
		(95%CI)		(95%CI)		(95%CI)		(95%CI)		(95%CI)		(95%CI)
**Red deer**	17/53	32.1	163/305	53.4	187/283	66.1	160/366	43.7	197/349	56.4	724/1356	**53.4^a^**
		(19.5–44.7)		(47.8–59.0)		(60.6–71.6)		(38.6–48.8)		(51.2–61–6)		**(50.7–56.0)**
**Fallow deer**	0/43	0	21/44	47.7	16/52	30.8	14/88	15.9	-	-	51/227	**22.4^b^**
				(32.9–62.5)		(18.2–43.3)		(8.3–23.5)				**(17.0–27.8)**
**Roe deer**	0/1	0	9/23	39.1	15/32	46.9	22/43	51.2	17/31	54.8	63/130	**48.5^a^**
				(19.2–59.0)		(29.6–64.2)		(36.3–66.1)		(37.3–72.3)		**(39.9–57.1)**
**Spanish ibex**	-	-	3/13	23.1	0/20	0	1/3	33.3	-	-	4/36	**11.1^b^**
				(0.2.46.0)				(0–86.6)				**(0.8–21.4)**
**European** **mouflon**	0/1	0	-	-	3/4	75.0	8/11	72.7	6/15	40.0	17/31	**54.8^a^**
						(32.6–117.4)		(46.4–99.0)		(15.2–64.8)		**(37.3–72.3)**
**Cantabrian** **chamois**	31/42	73.8	16/47	34.0	18/28	64.2	5/13	38.4	0/1	0.0	70/131	**53.4^a^**
		(60.5–87.1)		(20.5–47.5)		(46.4–81.9)		(12.0–64.8)				**(44.8–61.9)**
**Aoudad**	-	-	-	-	1/2	50.0	1/1	100.0	-	-	2/3	**66.7^ab^**
						(0–119.3)		(0–68.8)				**(13.4–120.0)**
**Total**	48/140	34.3	212/432	49.1	240/421	57.0	211/525	40.2	220/396	55.5	931/1914	**48.6**
		(26.4–42.2)		(44.4–53.8)		(52.3–61.7)		(36.0–44.4)		(50.6–60.4)		**(46.4–50.8)**

Dashes (−) indicate no samples available. Superscripts (a,b) indicate statistically significant differences (*P*<0.05) in prevalence among species.

Other statistical analyses than the pure autoregressive models were performed using SPSS 20.0 (Statistical Package for Social Sciences (SPSS) Inc., Chicago, IL, USA). Differences were considered statistically significant when *P*-value <0.05.

## Results

### Descriptive Statistics

A total of 931 out of the 1,914 (48.6%, 95% CI: 46.4–50.8) serum samples analyzed by ELISA were positive to BTV. Seropositivity was detected in all years, in all wild ruminant species analyzed, and in all sampled bio-regions (Tables 1 and 2). Statistically significant differences were observed among species: red deer, roe deer, European mouflon and southern chamois showed significantly higher seroprevalence by ELISA than fallow deer and Spanish ibex (*P*<0.05) (Table 1). No statistically significant differences were observed between age classes (*P* = 0.071) and between males and females (*P* = 0.235). Specific seroprevalence by each serotype and bio-region is shown in Table 2.

**Table 2:** Seroprevalence (%) of each serotype by bio-region analyzed by means of serum neutralisation test (SNT) and RT-qPCR results.

	Seroprevalence (SNT positive/(SNT analysed + ELISA negative))				
	Bio-region 1	Bio-region 2	Bio-region 3	Bio-region 4	Total
**BTV-1**	43/351^a^	5/188^b^	114/924^a^	2/153^b^	**164/1616**
% (95% CI)	12.2 (8.8–15.6)	2.7 (20.3–32.9)	12.3 (10.2–14.5)	1.3 (7.7–18.4)	**10.5 (8.7–11–6)**
**BTV-4**	0/351^a^	0/188^a^	127/924^b^	127/153^b^	**149/1616**
% (95% CI)	0	0	13.7 (11.5–15.9)	83.0 (77.0–88.9)	**9.2 (7.8–10.6)**
**BTV-8**	0/351^a^	0/188^a^	3/924^a^	0/153^a^	**3/1616**
% (95% CI)	0	0	0.3 (0–0.7)	0	**0.2 (0–0.4)**
**RT-qPCR**	18/40	0/15	14/246	0/10	**32/311**

Superscripts (a, b) indicate statistically significant differences (*P*<0.05) among bio-regions for each serotype. Cytotoxic ELISA positive sera (N = 289) were not analyzed by SNT.

BTV RNA was detected in 32 out of the 311 spleen samples analyzed (10.3% 95% CI: 7.0–13.6), 18 belonging to bio-region 1 (Atlantic) and 14 to bio-region 3 (South-Central) (Table 2). BTV RNA was detected in red (28/257) and fallow deer (4/40), whereas all the samples from other species (N = 25) were negative (*Ct*>40) to RT-qPCR.

**Figure 2:**
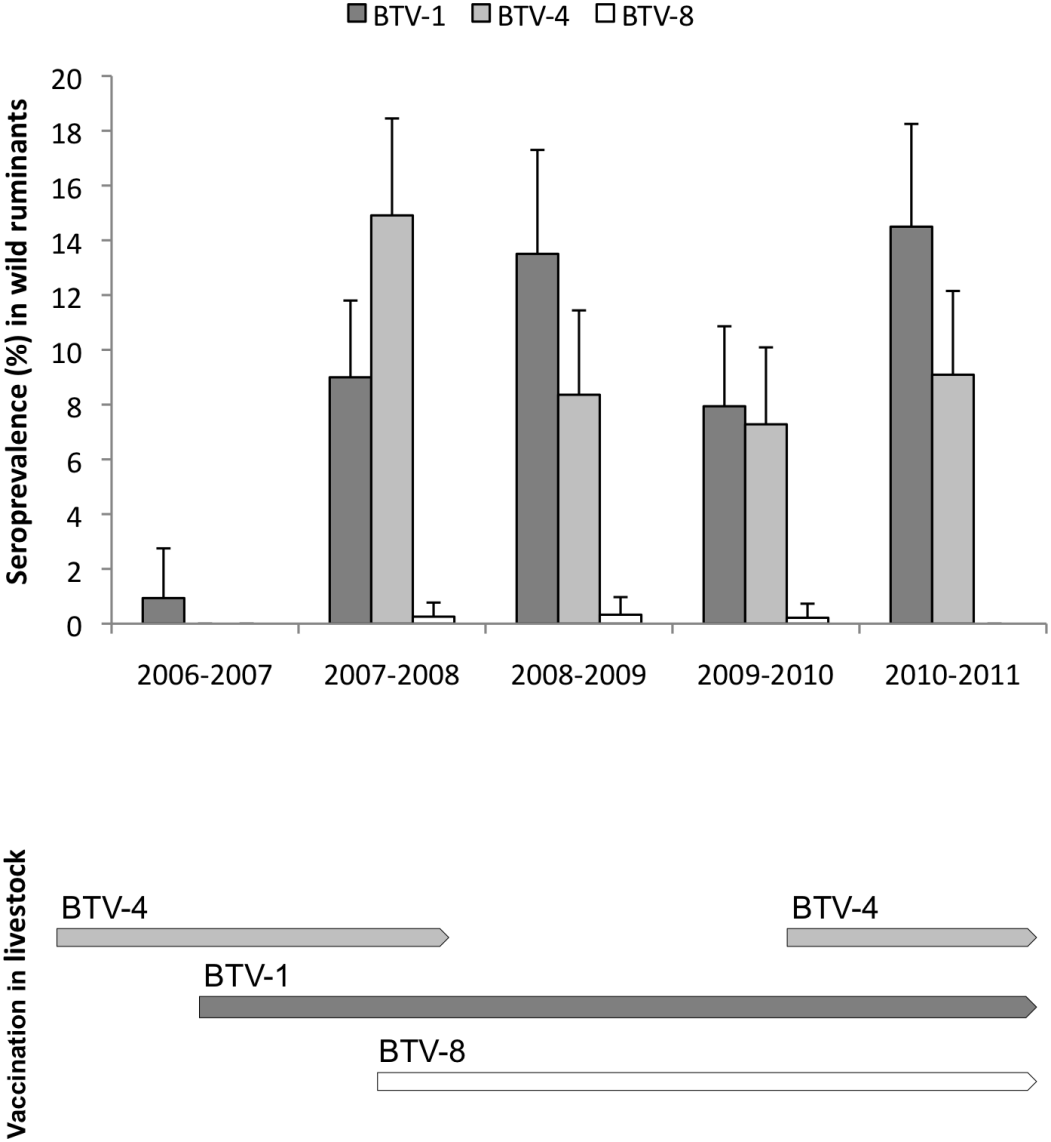
Temporal distribution of seropositve samples: from wild ruminants analysed by serumneutralisation test (upper graph) and representation of compulsory vaccination periods in livestock from Spain per each BTV serotype (bottom graph).

Five out of the 18 BTV RNA positive samples from bio-region 1 were positive to BTV-1 by SNT, the remaining 13 positive samples from this bio-region were not available for SNT due to cytotoxicity of the sera. All RT-qPCR positive samples from bio-region 3 came from southern areas (Sierra Morena). Seropositivity to each BTV serotype by yearly sample period is shown in Figure 2. A total of 227 ELISA positive samples could not be interpreted by SNT due to cytotoxicity of sera. Seroprevalence was calculated as the ratio of SNT-positive samples to SNT-analyzed samples plus ELISA-negative samples (cytotoxic samples were not included).

### Spatial Epidemiology: Relationships between Livestock and Wild Ruminants

**Figure 3:**
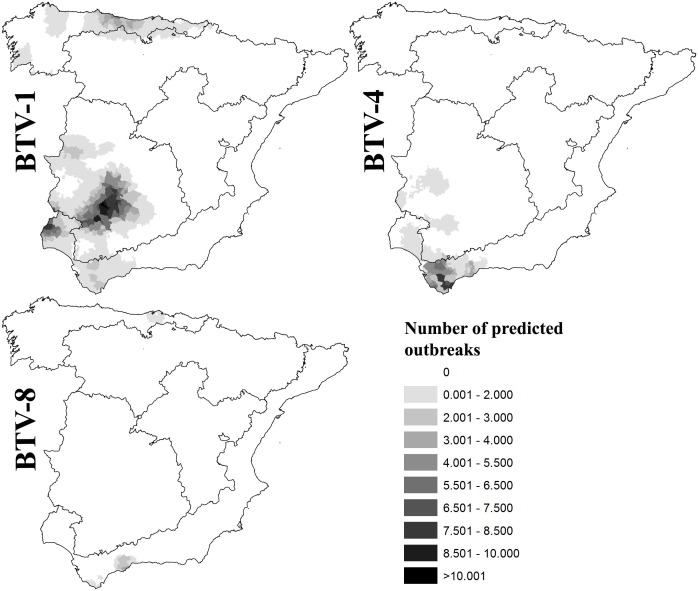
Spatial patterns of BT outbreaks in livestock at the municipality level during 2003–2012 according to the predictions from the autoregressive models. Independent patterns for each BTV serotype are shown. Number of seropositive/analysed samples by serumneutralisation test (SNT) are shown in each bio-region.

The spatial patterns of BT outbreaks for each BTV serotype according to the predictions from autoregressive models are shown in Figure 3. The residuals of these models were spatially independent according to Moran’s I test (*I* = 0.026, *I* = 0.044 and *I* = 0.043; for the BTV-1, BTV-4 and BTV-8 models, respectively). The R^2^ values were 0.384, 0.242 and 0.543, for the BTV-1, BTV-4 and BTV-8 models, respectively.

The number of predicted outbreaks in livestock and the relative abundance of red deer were positively related to BTV-1 detection in red deer (see Table 3). The interaction of these predictors was negatively and significantly related to the response variable, suggesting that the strength of the relationship between outbreaks in livestock and BTV prevalence in red deer decreases as red deer abundance increases.

**Table 3:** Summary of models used to explore the epidemiological relationship between the number of BT outbreaks in livestock (as predicted from an autoregressive model) and prevalence on red deer populations.

AICc	BT outbreaks in livestock (BTO)	Red deer relative abundance (RED)	BTO*RED
373.26	32.62**	8.81**	(−) 6.45*
378.03	67.11**	3.75*	
379.98	65.57**		

Akaike information criteria for small samples (AICc; [48]) is reported to compare between model (better as AICc decrease). Wald statistic and p-values (*P<0.05, **P<0.01).

## Discussion

Antibodies against BTV-1, BTV-4, and BTV-8 were detected in wild ruminant species in spite of ongoing vaccination campaigns in domestic livestock during the study period (2006–2011), agreeing with previous results in the Iberian Peninsula [11], [14], [17]. Overall, distribution of BTV serotypes in wild ruminants matched distribution of serotypes detected in domestic ruminants. In this study, BTV-1 was detected mainly in Atlantic (1) and South-Central (3) bio-regions where this serotype has been detected in livestock [19], and BTV-4 was found in South-Central (3) and Interior mountains (4) bio-regions. The major part of the BTV-4 outbreaks in livestock have been reported in Southern Spain, but in the Interior mountains (4) bio-region no BTV-4 outbreaks were reported in domestic livestock during the study period [19]. Therefore, BTV-4 was again detected in wild ruminants in regions where no outbreaks in domestic livestock occurred [17]. Moreover, in South-Central (3) bio-region seroprevalence against BTV-1 and BTV-4 in wild ruminants was similar, although BTV-1 has been detected in livestock more frequently than BTV-4, further suggesting that BTV prevalence in wild ruminants might not be always correlated with outbreaks in livestock. The spatial analysis revealed that at high red deer abundances, livestock outbreaks and BTV seroprevalence in red deer are less related, which suggests that once established BTV dynamics in abundant red deer populations could be more independent from BTV cycle in domestic livestock than when BTV enters low abundant red deer populations. The detection in domestic or sentinel animals of BTV outbreaks out of the main vector season (February) in Interior-mountains (4) bio-region in 2009–2010 could probably be related to a reversion from BTV self-maintaining wild ruminants to domestic livestock rather than to an unlikely arrival of new vectors out of season. This agrees with previous publications which suggest red deer as a maintenance or even as a potential reservoir host, with other species acting as dead-end hosts due either to population traits or to species-specific BTV infection related factors [16], [17], [23], [42]. There is still a great gap in knowledge on wild ungulate-biting midge relationships and their implication in BTV life-cycle that needs to be targeted in the future to properly understand the implication of wild ungulates in BTV ecology.

Vaccination-induced antibodies against BTV may last from 90 days to three years, depending on BTV serotype, analytical methodology (ELISA or SNT) and individual factors [43], [44]. Clearance of protective neutralising antibodies in domestic livestock after the end of compulsory vaccination programs, combined with BTV circulation within the unvaccinated red deer populations, could lead to new BTV re-infection episodes of non-protected livestock, as the ones suspected in Interior-mountains (4) bio-region in 2009–2010. Moreover, since BTV dynamics in red deer could be independent of the domestic livestock BTV cycle, the abundant wild free-ranging red deer populations could be used as sentinel for BT more reliably than the limited number of unvaccinated domestic ruminants kept in sentinel farms, as shown by BTV detection in red deer in regions where no BT outbreaks had been declared in livestock and suggested in previous publications [10], [12], [14], [17], [22], [27], [45]. Nevertheless, in spite of the potential implication of wild ruminants, especially red deer, in the maintenance and potential transmission of BTV, BTV infection from wild ruminants to domestic livestock is still to be proven.

## Conclusions

To summarize, our results provide the following evidences on the role of wild ungulates in BTV epidemiology in Spain: i) BTV-1, BTV-4, and BTV-8 antibodies were detected in wild ruminant populations in Spain during the 2006–2011 period in spite of ongoing BTV vaccination campaigns in domestic livestock; ii) BTV dynamics in wild ruminant populations from Spain may gain independence from BTV domestic cycles, particularly when BTV enters abundant red deer populations, as indicated by the detection of BTV in wild ruminants from areas where BTV outbreaks have not been reported in domestic livestock and by spatial modeling results; and iii) due to its abundance, wide geographic distribution, and to its susceptibility to long-viraemic asymptomatic BTV infection, red deer could be useful as BTV sentinel, but could also constitute a source for BTV infection for domestic livestock once vaccine-induced immunity decreases after the end of compulsory vaccination campaigns.
